# Supplementary material for: Changes of In Situ Prokaryotic and Eukaryotic Communities in the Upper Sanya River to the Sea over a Nine-Hour Period
Source: Microorganisms. 2023 Feb 20;11(2):536. doi: 10.3390/microorganisms11020536 (PMC9964997; doi:10.3390/microorganisms11020536)
Supplement: Supplementary file 1 [file microorganisms-11-00536-s001.zip › microorganisms-2156204-supplementary.pdf]

Supplementary Materials.

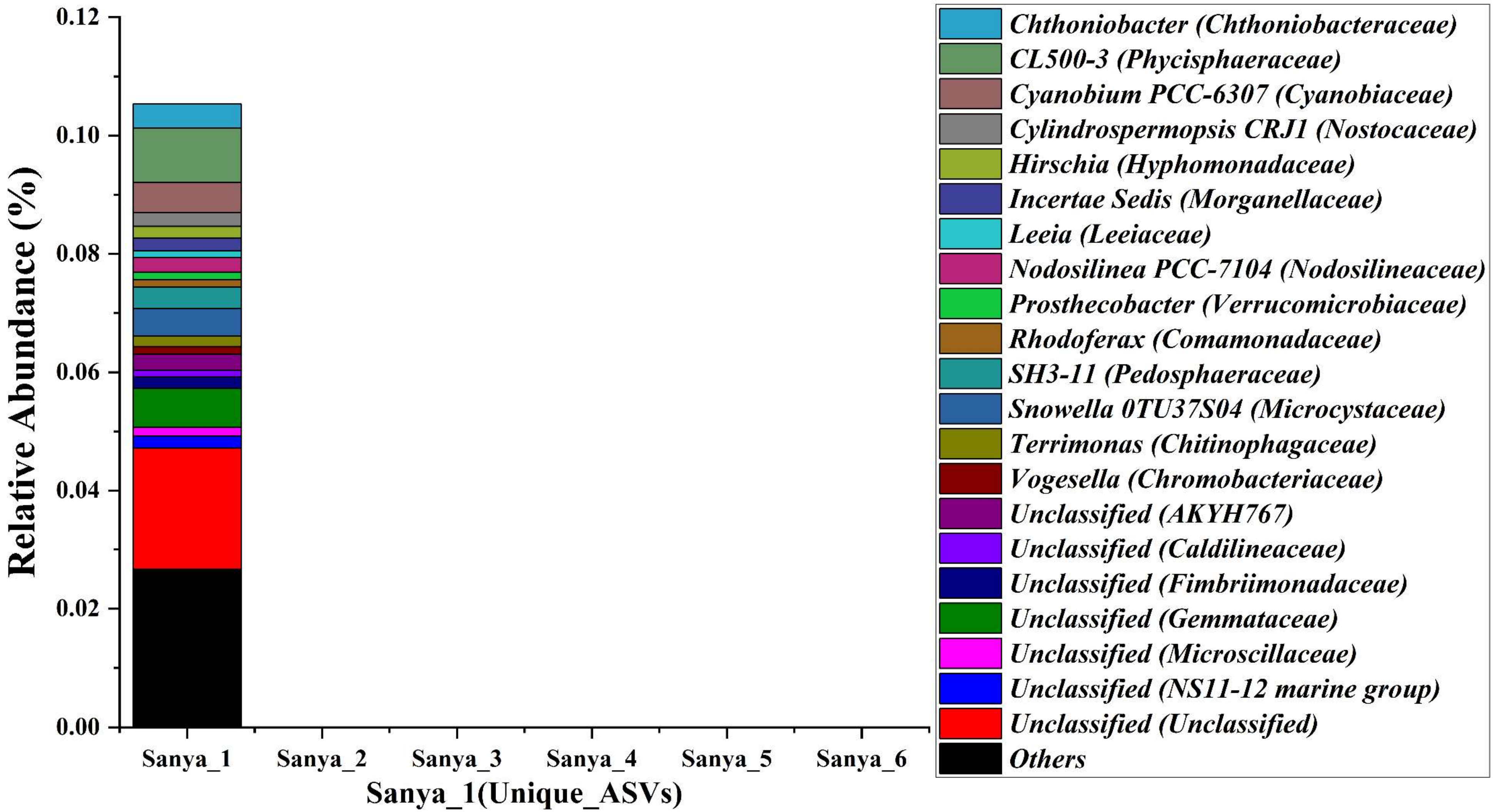

Figure S1. Distribution and composition of unique prokaryotic ASVs generated from Sanya 1.

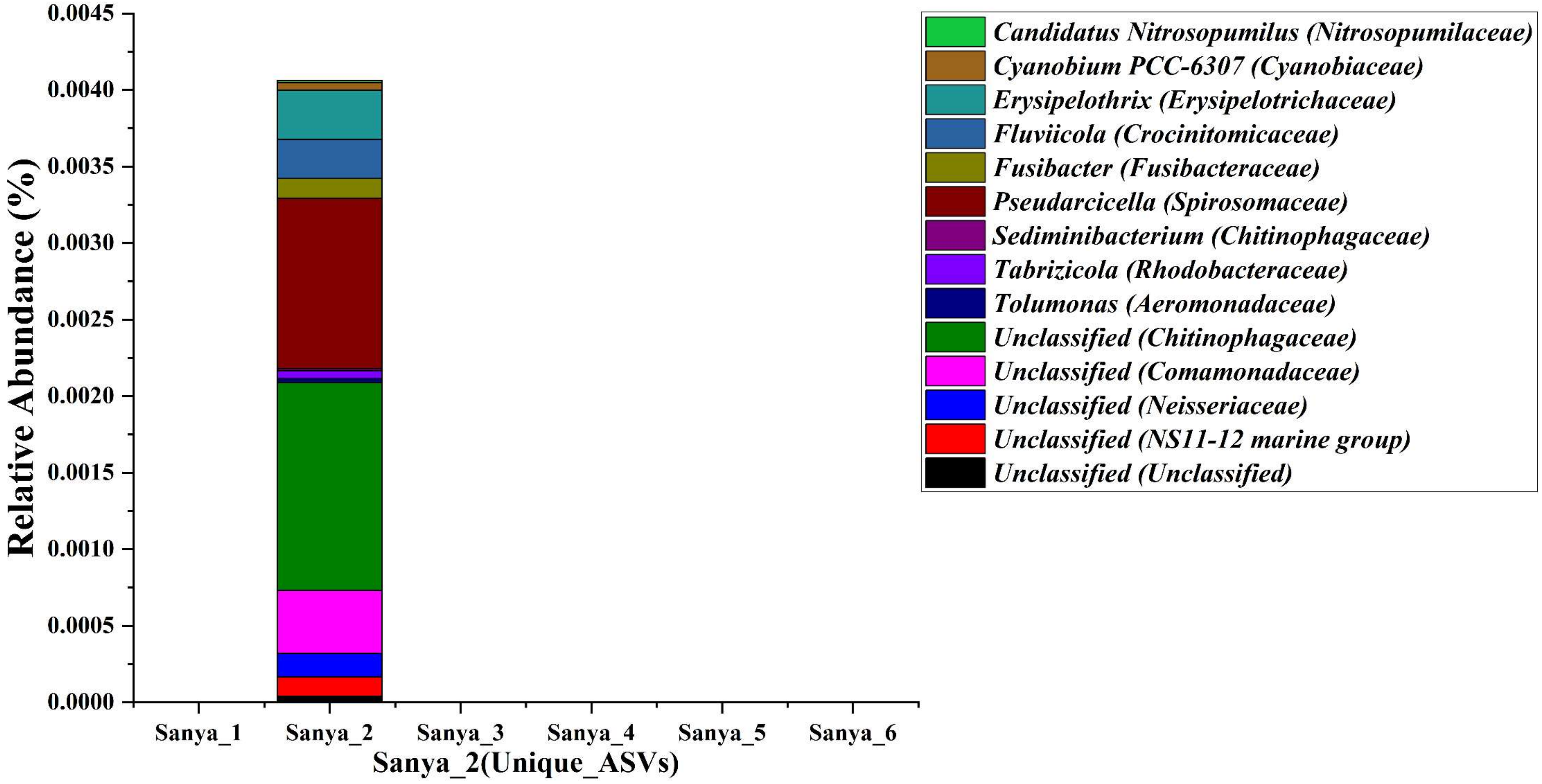

Figure S2. Distribution and composition of unique prokaryotic ASVs generated from Sanya 2.

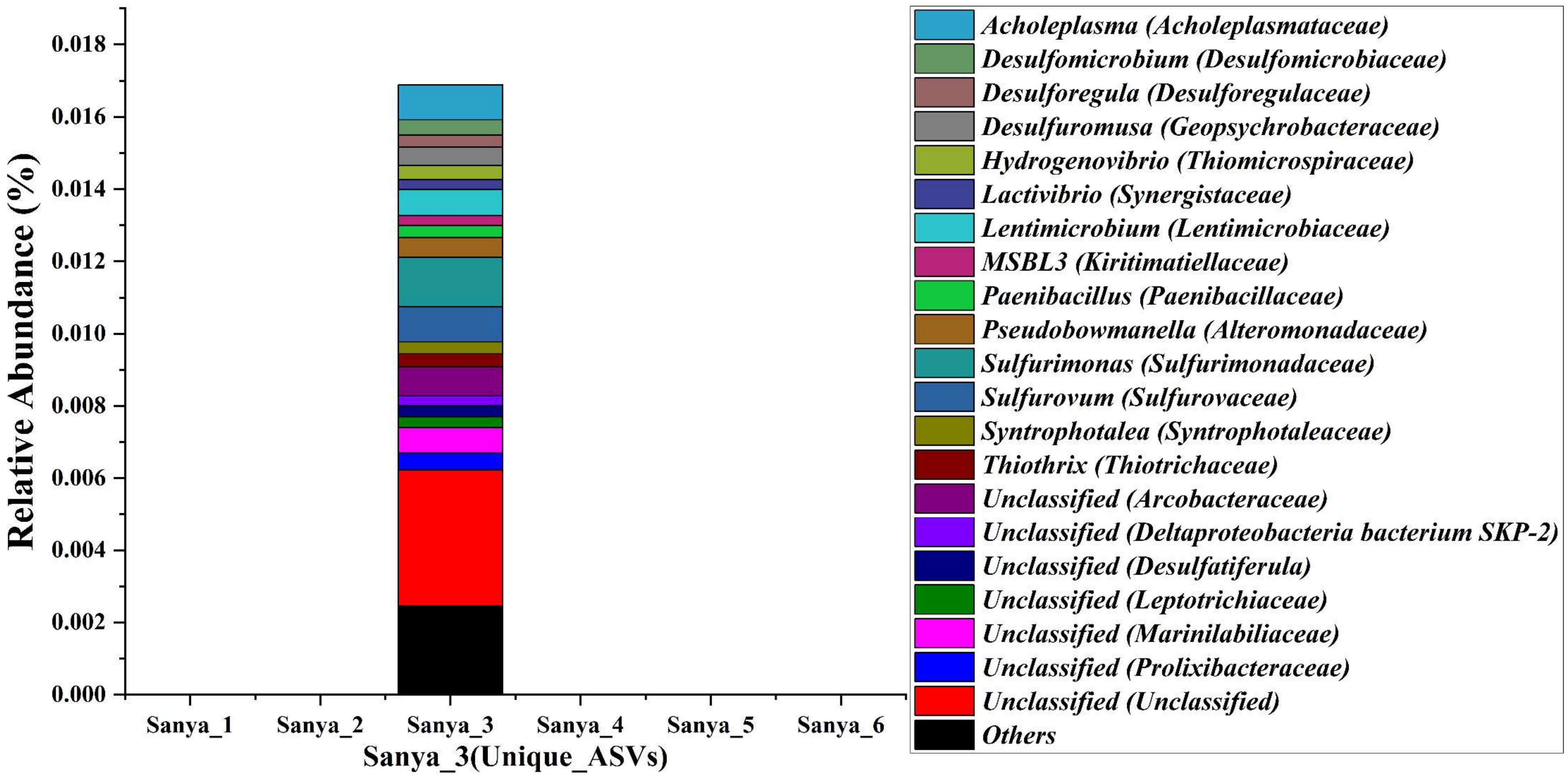

Figure S3. Distribution and composition of unique prokaryotic ASVs generated from Sanya 3.

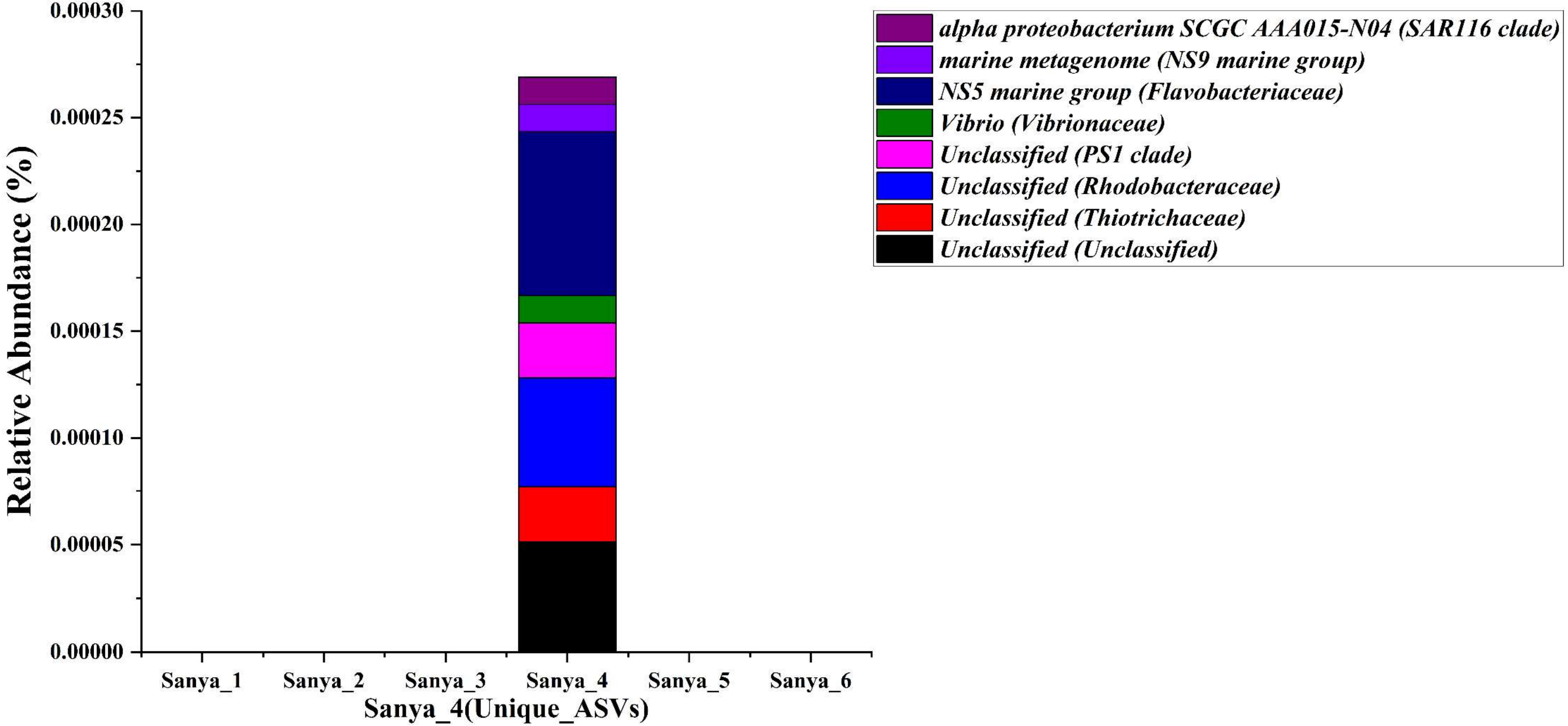

Figure S4. Distribution and composition of unique prokaryotic ASVs generated from Sanya 4.

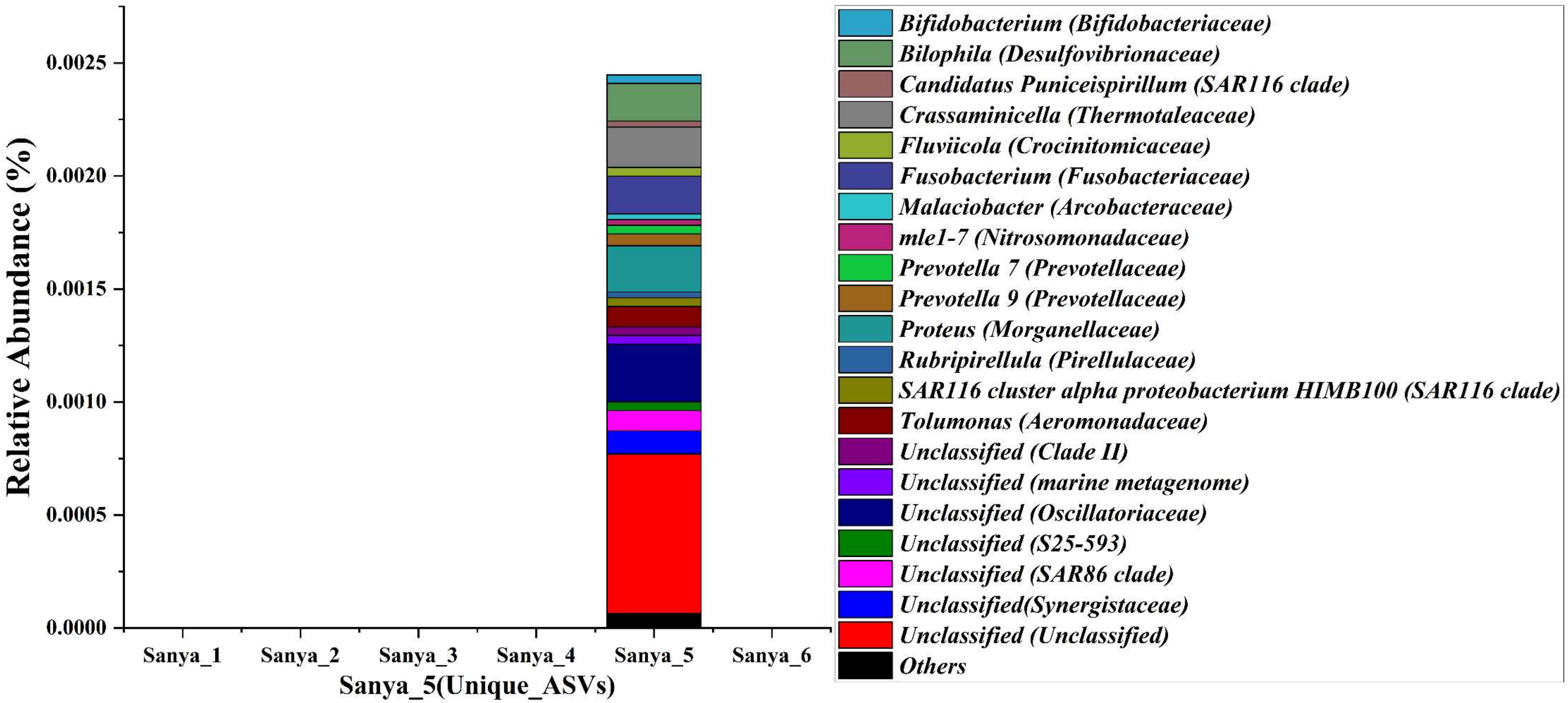

Figure S5. Distribution and composition of unique prokaryotic ASVs generated from Sanya 5.

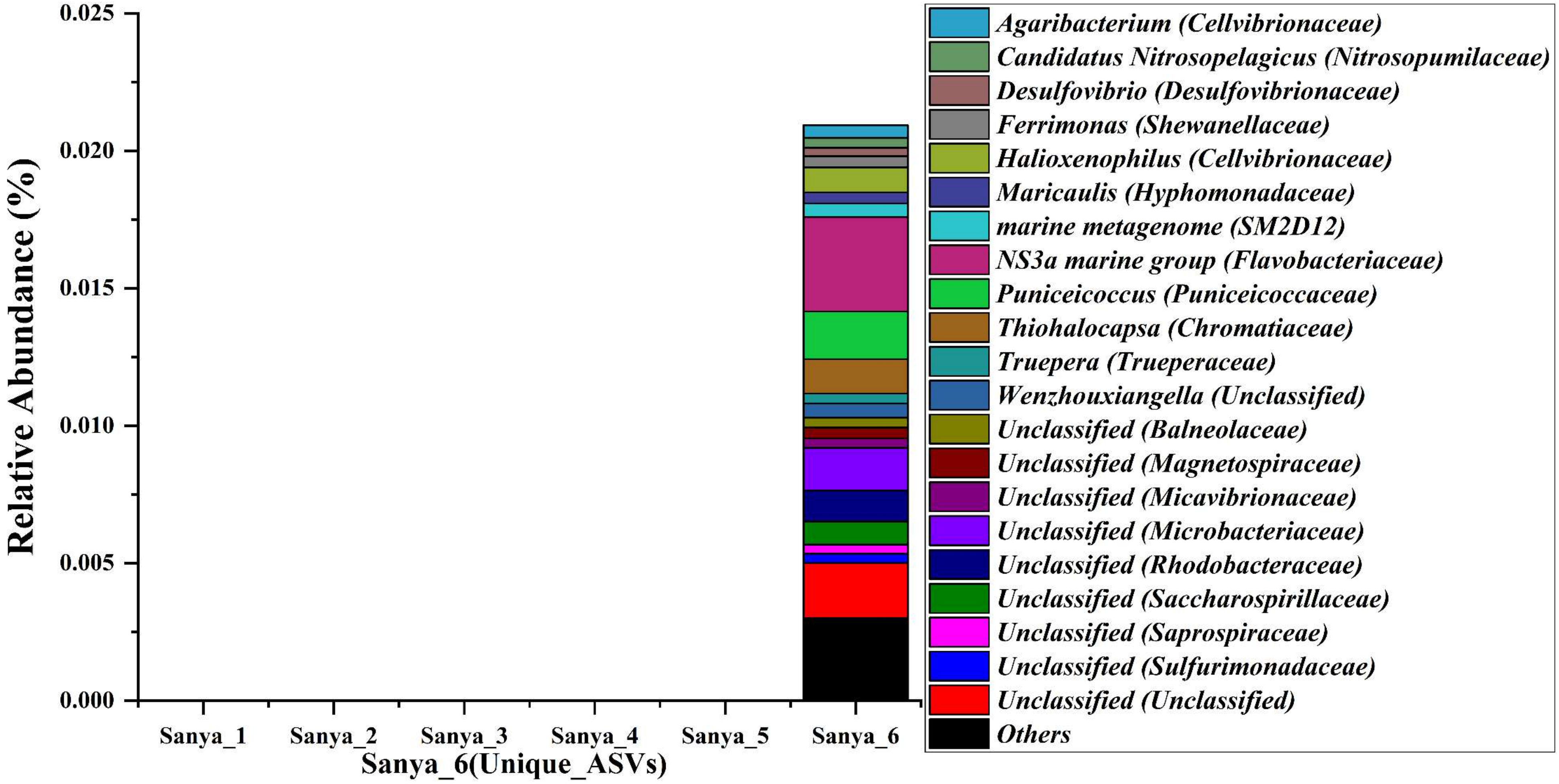

Figure S6. Distribution and composition of unique prokaryotic ASVs generated from Sanya 6.

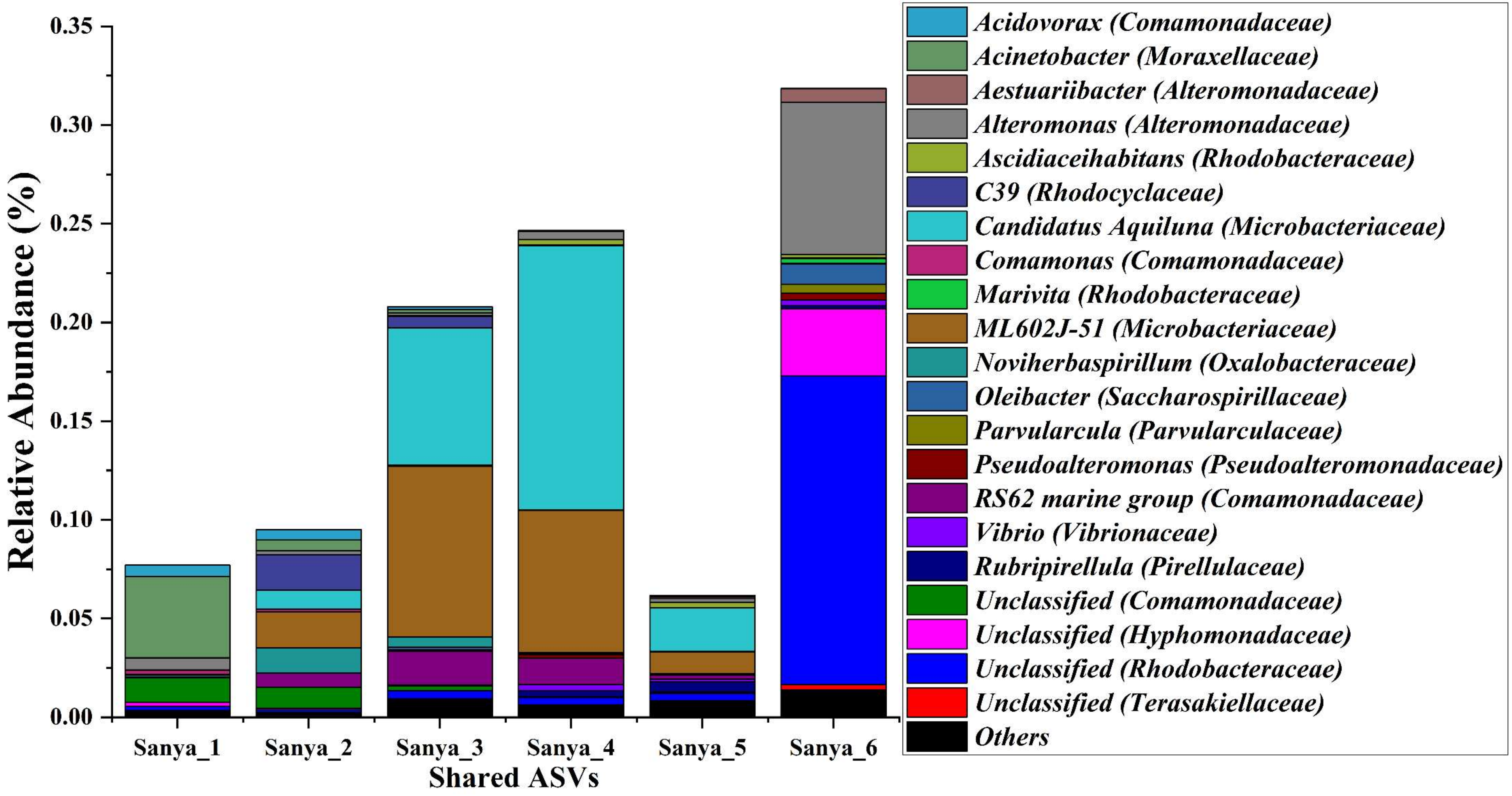

Figure S7. Distribution and composition of shared prokaryotic ASVs generated from all samples.

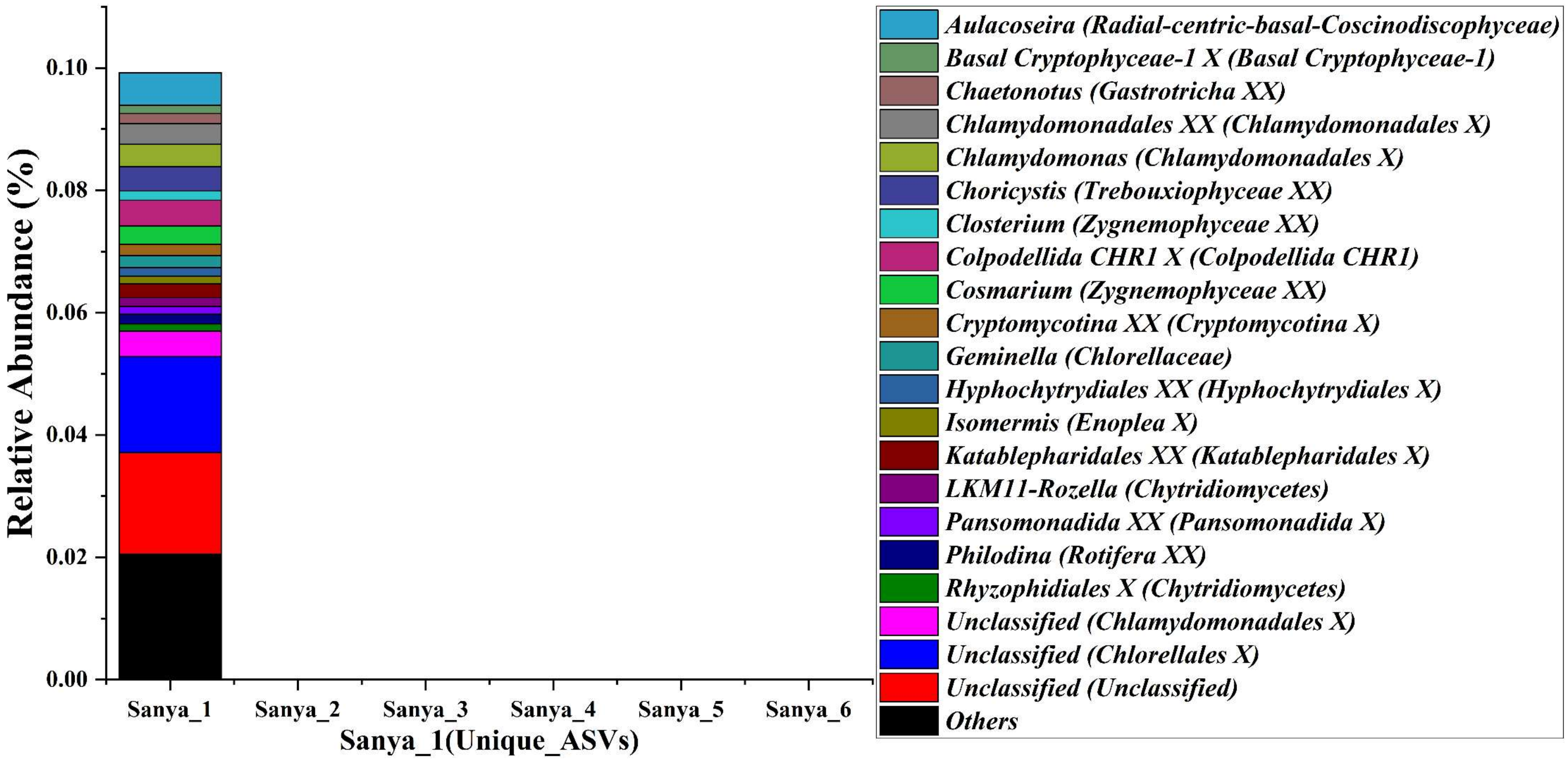

Figure S8. Distribution and composition of unique eukaryotic ASVs generated from Sanya 1.

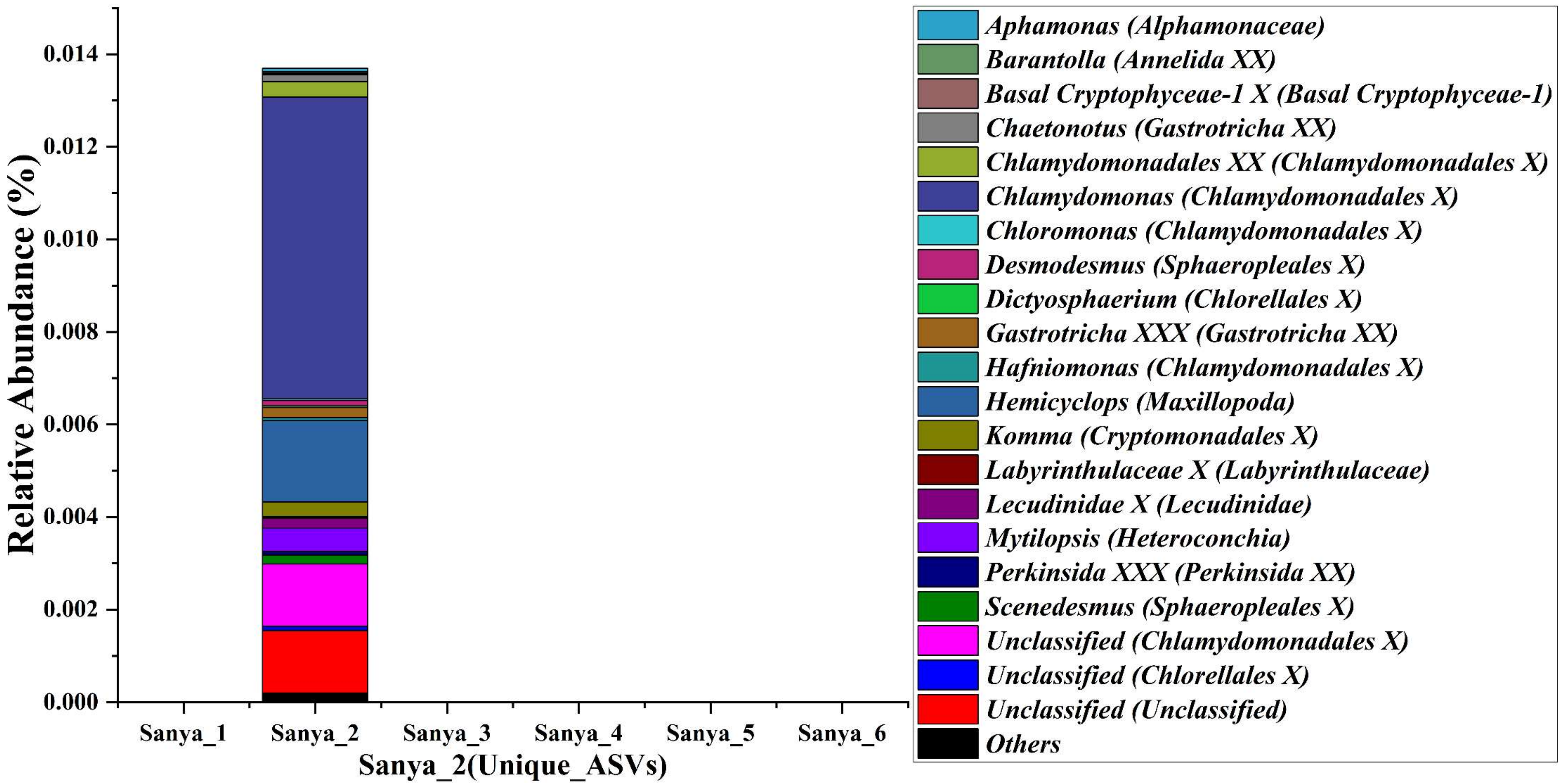

Figure S9. Distribution and composition of unique eukaryotic ASVs generated from Sanya 2.

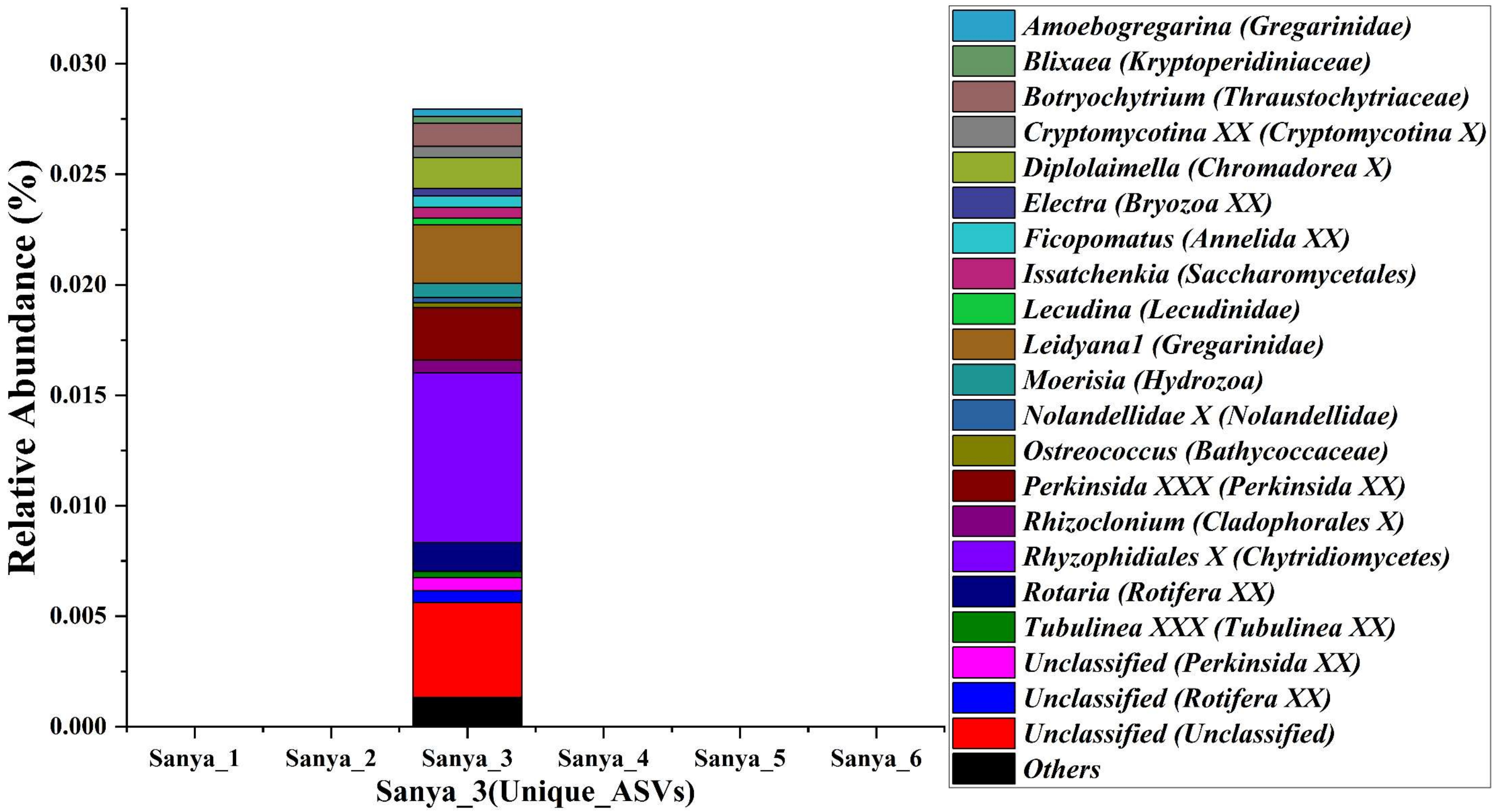

Figure S10. Distribution and composition of unique eukaryotic ASVs generated from Sanya 3.

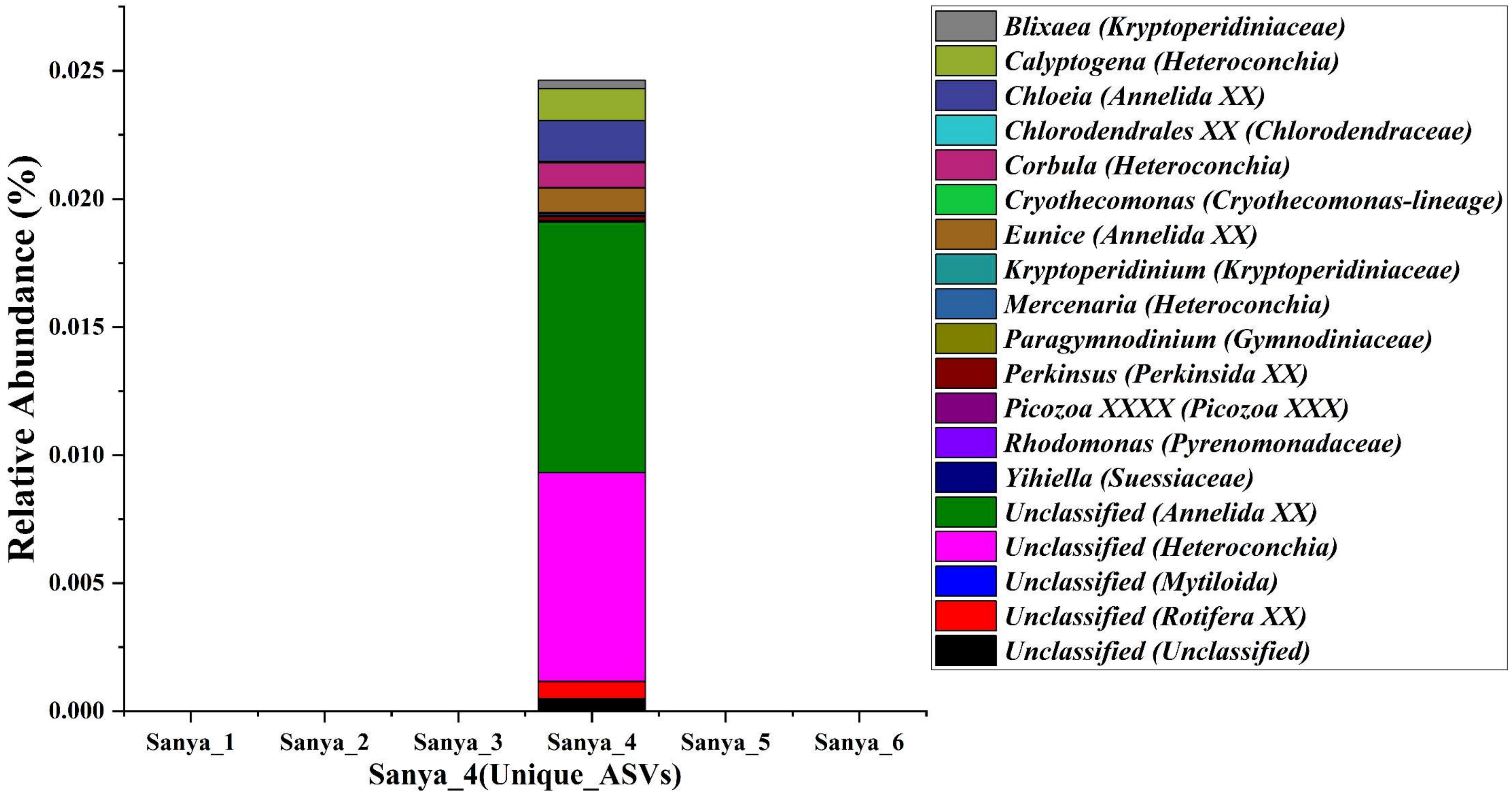

Figure S11. Distribution and composition of unique eukaryotic ASVs generated from Sanya 4.

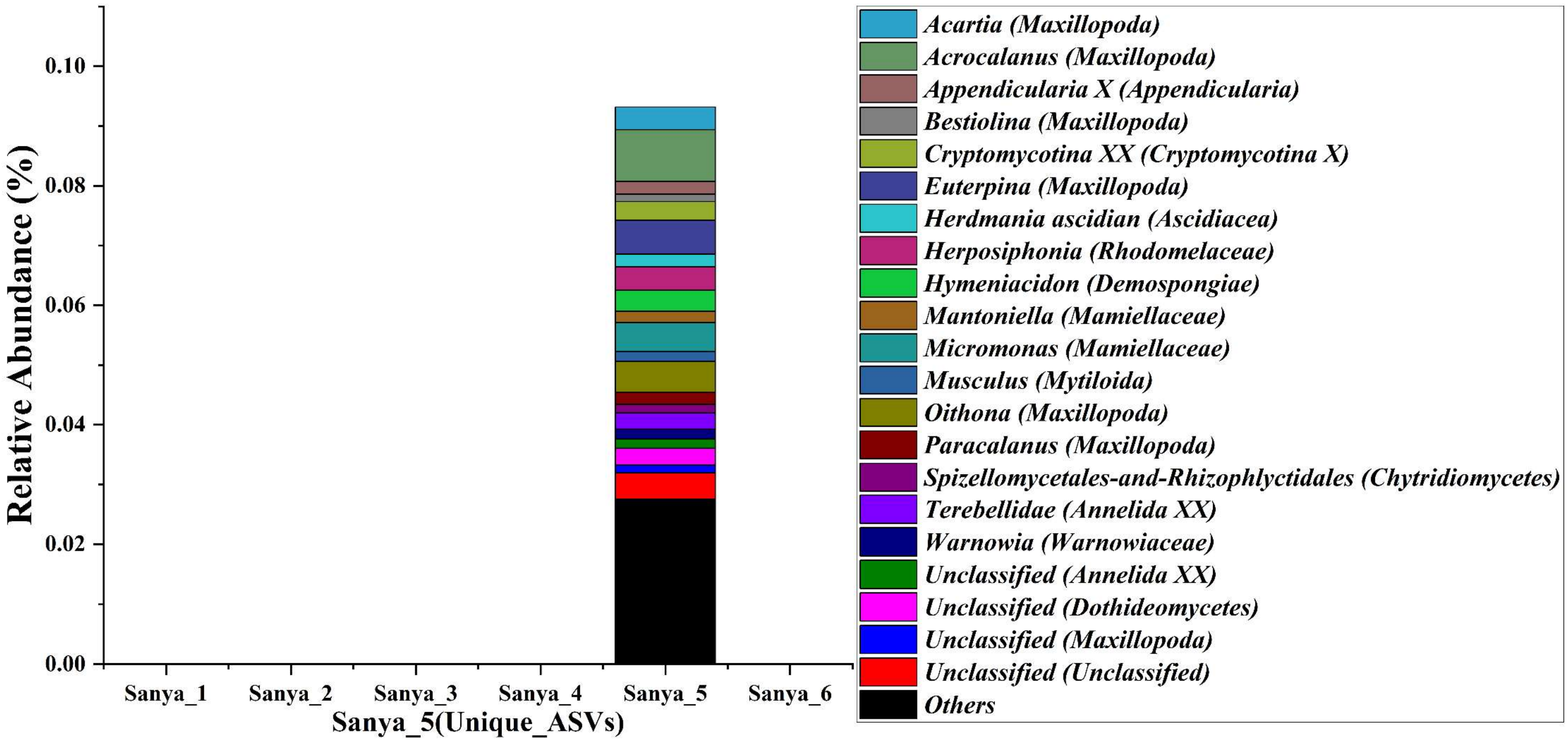

Figure S12. Distribution and composition of unique eukaryotic ASVs generated from Sanya 5.

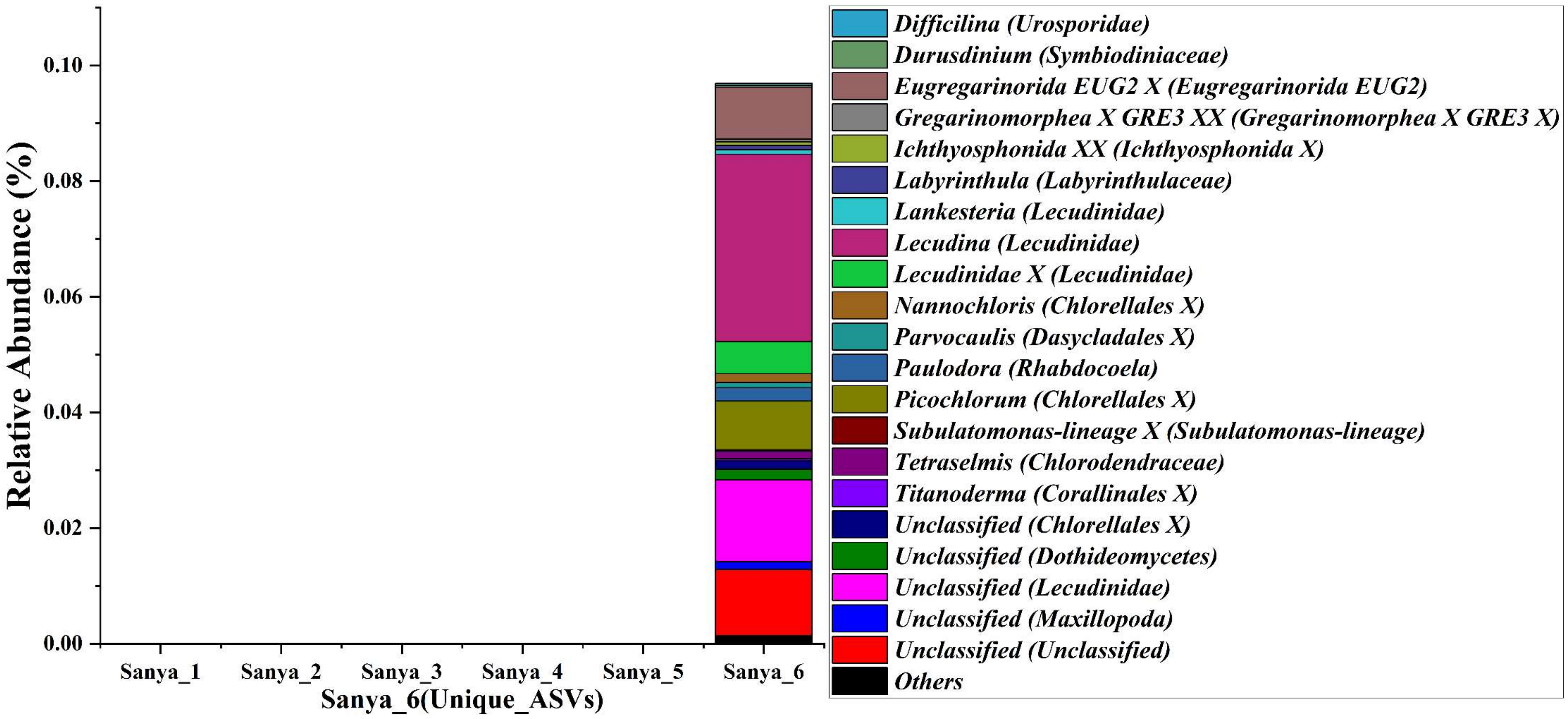

Figure S13. Distribution and composition of unique eukaryotic ASVs generated from Sanya 6.

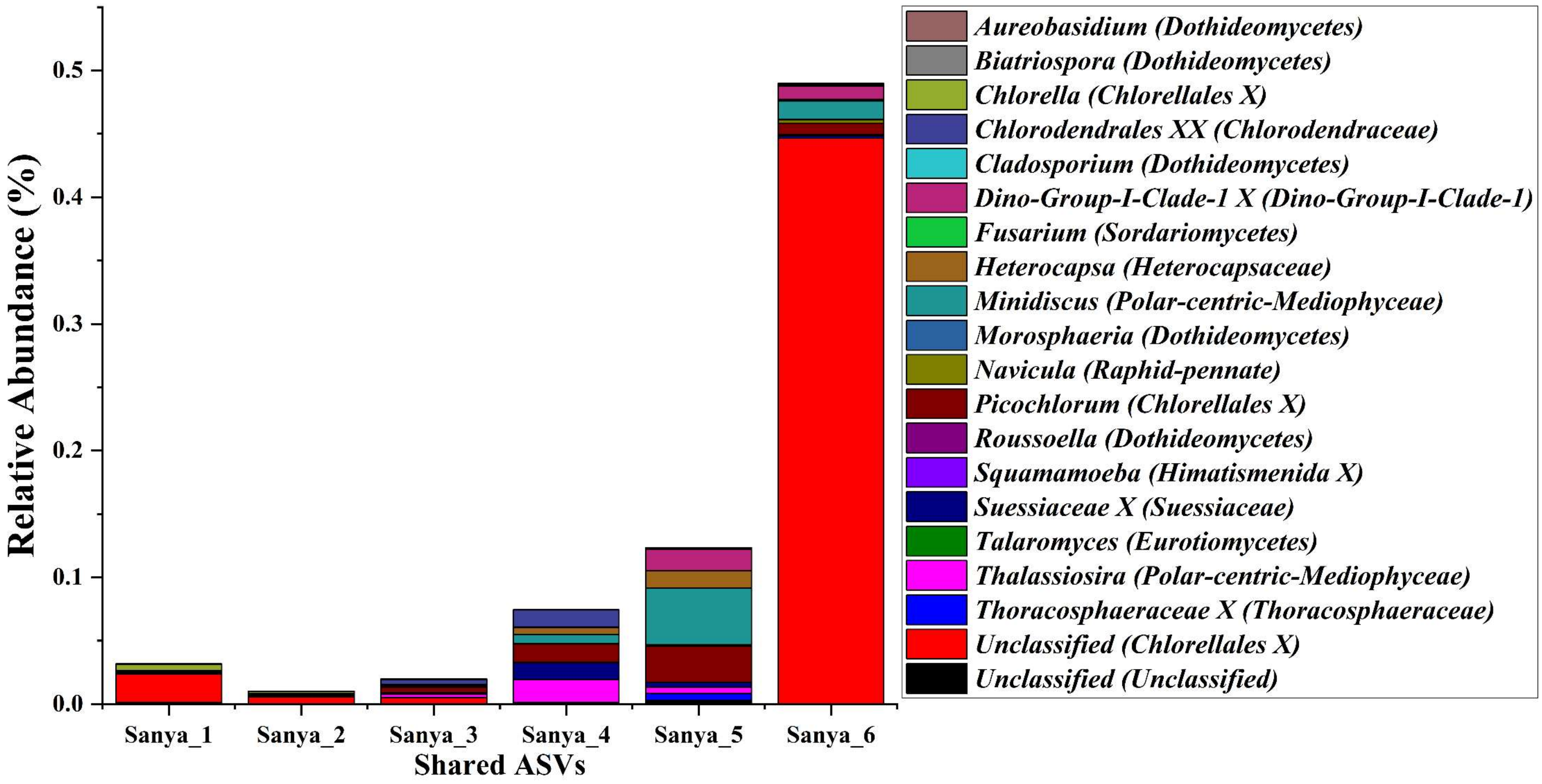

Figure S14. Distribution and composition of shared eukaryotic ASVs generated from all samples.
